# Accuracy of imaging in discriminating complicated from uncomplicated appendicitis in daily clinical practice

**DOI:** 10.1007/s00384-022-04173-z

**Published:** 2022-05-18

**Authors:** Matthijs D. M. Bolmers, Wouter J. Bom, Jochem C. G. Scheijmans, Anna A. W. van Geloven, Marja. A. Boermeester, Willem A. Bemelman, Charles. C. van Rossem, on behalf of the SNAPSHOT collaborators, GJ Van Acker, B Akkermans, GJ Akkersdijk, GD Algie, JH Allema, CS Andeweg, N Appeldoorn, JG van Baal, CM den Bakker, SA Bartels, C van den Berg, B Boekestijn, FC den Boer, D Boerma, AL van den Boom, MC Boute, SA Bouwense, J Bransen, FA van Brussel, OR Busch, SM de Castro, HA Cense, C Croese, T van Dalen, I Dawson, E van Dessel, R Dettmers, N Dhar, FY Dohmen, KW van Dongen, P van Duijvendijk, RR Dulfer, BJ Dwars, JP Eerenberg, M van der Elst, E van den Ende, LM Fassaert, JT Fikkers, JW Foppen, EJ Furnee, FP Garssen, MF Gerhards, H van Goor, RR Gorter, JS de Graaf, LJ Graat, J Groot, AC van der Ham, JF Hamming, JT Hamminga, E van der Harst, J Heemskerk, A Heijne, JT Heikens, E Heineman, R Hertogs, E van Heurn, LC van den Hil, AG Hooftwijk, CC Hulsker, DR Hunen, MS Ibelings, JM Klaase, R Klicks, L Knaapen, RT Kortekaas, F Kruyt, S Kwant, SS Lases, T Lettinga, A Loupatty, RA Matthijsen, RC Minnee, B Mirck, L Mitalas, D Moes, AM Moorman, VB Nieuwenhuijs, GA Nieuwenhuizen, PD Nijk, JM Omloo, AG Ottenhof, HW Palamba, DL van der Peet, IT Pereboom, PW Plaisier, AP van der Ploeg, MH Raber, MM Reijen, H Rijna, C Rosman, RM Roumen, RF Scmitz, APS van der Velden, WH Scheurs, TA Sigterman, HJ Smeets, DJ Sonnevled, MN Sosef, SF Spoor, LP Stassen, L van Steensel, E Stortelder, J Straatman, HJ van Susante, DES de Hoog, CT van Scheltinga, BR Toorenvliet, PC Verbeek, M Verseveld, JH Volders, MR Vriens, PW Vriens, BC Vrouenraets, BJ van de Wall, JA Wegdam, E Westerduin, JJ Wever, NA Wijfels, BP Wijnhoven, TA Winkel, DC van der Zee, AM Zeillemaker, C Zietse

**Affiliations:** 1 Department of Surgery, Dijklander Hospital, Hoorn, The Netherlands; 2 Department of Surgery, UMC, Location AMC, Amsterdam Gastroenterology & Metabolism, University of Amsterdam, Amsterdam, The Netherlands; 3 Department of Surgery, Tergooi MC, Hilversum, The Netherlands; 4 Department of Surgery, Maasstad Ziekenhuis, Rotterdam, The Netherlands

**Keywords:** Complicated appendicitis, Uncomplicated appendicitis, Imaging, Diagnostic accuracy

## Abstract

**Background:**

Radiologic imaging can accurately diagnose acute appendicitis, but little is known about its discriminatory capacity between complicated and uncomplicated appendicitis.

**Objective:**

This study aims to investigate the accuracy of imaging in discriminating complicated from uncomplicated appendicitis.

**Methods:**

Data was used from the prospective, nationwide, observational SNAPSHOT appendicitis database, including patients with suspected acute appendicitis who were planned for an appendectomy. Usage of ultrasound (US), CT, MRI or a combination was recorded. Radiological reports were used to group for complicated or uncomplicated appendicitis. The reference standard was based on operative and pathological findings. Primary outcomes were sensitivity and specificity in discriminating complicated from uncomplicated appendicitis. Secondary outcomes were diagnostic accuracy results per imaging modality and for the subgroups age, BMI, and sex.

**Results:**

Preoperative imaging was performed in 1964 patients. In 1434 patients (73%), only US was used; in 109 (6%) patients, only CT was used; and 421 (21%) patients underwent US followed by CT or MRI. Overall, imaging workup as practiced, following the national guideline, had a poor sensitivity for complicated appendicitis of only 35%, although specificity was as high as 93%. For US, accuracy for complicated appendicitis was higher in children than in adults; sensitivity 41.2% vs. 26.4% and specificity 94.6% vs. 93.4%, respectively, *p* = 0.003. For relevant subgroups such as age, sex and BMI, no other differences in the discriminatory performance were found.

**Conclusion:**

A diagnostic workup with stepwise imaging, using a conditional CT or MRI strategy, poorly discriminates between complicated and uncomplicated appendicitis in daily practice.

**Supplementary information:**

The online version contains supplementary material available at 10.1007/s00384-022-04173-z.

## Introduction

According to the current standard of practice, the use of imaging in the workup for acute appendicitis leads to a decrease in the negative appendectomy rate [1–3]. In the Netherlands, this workup mainly includes an ultrasound (US) followed by a conditional CT scan (CT) in case of negative or inconclusive US, or in children, young adults and pregnant women an MRI [2, 4]. All imaging modalities are subjected to their availability and accuracy. Besides that, they may have specific disadvantages like radiation.

There is a growing belief that uncomplicated and complicated appendicitis, or simple and complex appendicitis, are two different entities [5]. The presence of necrosis indicates the major difference between uncomplicated and complicated appendicitis. Complicated appendicitis is defined by the presence of necrosis. It is thought that uncomplicated appendicitis does not develop necrosis, and, therefore, will not progress into complicated appendicitis [6]. On the contrary, it could be hypothesised that patients with complicated appendicitis present with complicated appendicitis from the start of the disease.

It is relevant to discriminate complicated from uncomplicated appendicitis. For uncomplicated appendicitis, recent studies suggest that uncomplicated appendicitis may be treated with antibiotics alone [7–9]. Although effective and safe, this conservative treatment has a risk of recurrent appendicitis, increasing to 40% after 5 years [10]. On the other hand, patients with complicated appendicitis should not be treated with antibiotics alone because of the chance of perforated appendicitis. Guidelines advise to perform surgery for patients with complicated appendicitis as soon as possible, or at least within 8 h after diagnosis [1, 11].

These differences in the treatment regimen make it essential to recognise and treat complicated appendicitis within 8 h when patients present to the hospital.

Some studies have described the diagnostic accuracy of discriminating between complicated and uncomplicated appendicitis for the imaging workup [12, 13]. Others used a scoring system, including clinical features combined with radiological features [14, 15]. These studies were mostly setup as diagnostic accuracy studies in which operators were aware of study participation. We conducted an audit in which imaging results were collected in all patients operated for acute appendicitis in order to describe the accuracy of different imaging strategies in both uncomplicated and complicated appendicitis. This study aims to investigate the diagnostic accuracy of imaging in discriminating complicated from uncomplicated appendicitis in everyday practice.

## Materials and methods

### Study design

For this study data, from the SNAPSHOT appendicitis database was used. This database contains data from a prospective, nationwide, observational study, which included 1975 consecutive patients who underwent surgery for suspected appendicitis during 2 months in 62 Dutch hospitals (3 months in a pilot setting in eight hospitals). Patients who were treated conservatively by antibiotics or radiological drainage for suspected appendicitis were not included. Complete methods have been described previously [16].

### Data collection

Surgical residents scored clinical variables at the emergency department, and collected findings from imaging, surgical and histological reports. Data about the imaging modality were collected, as were imaging findings as interpreted by this physician. This interpretation was a diagnosis based on the imaging report and could include the following options: uncomplicated appendicitis, complicated/perforated appendicitis, acute appendicitis with an appendicular infiltrate/abscess or inconclusive. The radiology reports were not standardized and full reports were not collected in the database.

### Test methods

The index test was the interpretation of imaging findings and conclusions of the radiologist by the surgical resident. This interpretation is crucial for treatment decisions and is therefore representative for clinical practice. For the index test, complicated/perforated appendicitis or acute appendicitis with an appendicular infiltrate/abscess was classified as complicated appendicitis.

The reference standard was a final diagnosis of complicated appendicitis, uncomplicated appendicitis and no appendicitis based on surgical and histologic findings. Complicated appendicitis was defined as perforated or gangrenous appendicitis, or if antibiotics were required immediately after surgery. The group of patients whose final diagnosis was ‘no appendicitis’ included patients with an uninflamed appendix, a neoplasm of the appendix, or another diagnosis, according to the pathologist or surgeon.

### Outcomes

The primary outcome is the diagnostic accuracy in discriminating complicated from uncomplicated appendicitis for the imaging workup as performed in line with the national guideline. Sensitivity, specificity, positive predictive values (PPV) and negative predictive values (NPV) were calculated. Secondly, these values were described for US, primary CT, primary MRI and a fourth group, including US with conditional CT and US with conditional MRI. In an additional analyses, the reference standard, performed by surgeon only (based on perioperative findings) and pathologist only (based on histopathological findings), was analysed separately.

All outcomes were measured for the subgroups of adults vs. children and male vs. female patients. For patients older than 16, body mass index (BMI) was calculated and divided into subgroups BMI < 25 vs. ≥ 25.

As we only included patients who underwent appendectomy, no true negatives (TN) (patients correctly labelled as having no appendicitis) were available in this dataset. Therefore, no diagnostic accuracy measures for simply the diagnosis of appendicitis could be calculated. The focus of this study was discrimination between complicated and uncomplicated appendicitis.

#### Uncomplicated versus complicated appendicitis

To discriminate complicated from uncomplicated appendicitis, 3 × 3 Tables were constructed, comprising the diagnoses complicated appendicitis, uncomplicated appendicitis and no appendicitis. As it is in our interest to rule out complicated appendicitis, 2 × 2 contingency Tables were constructed out of 3 × 3 Tables. Therefore, patients with inconclusive outcomes were added to the group of expected uncomplicated appendicitis. Patients without primary appendicitis, according to the reference standard, were added to the reference group of uncomplicated appendicitis.

### Data analysis

IBM SPSS Statistics version 25.0 was used for analysis. As only descriptive outcomes were calculated, *X*^2^ was used for significant differences for sensitivity and specificity in the subgroups. In this case, only the lowest *p*-value was reported. A *p*-value < 0.05 was considered as statistically significant.

## Results

### Baseline

**Fig. 1:**
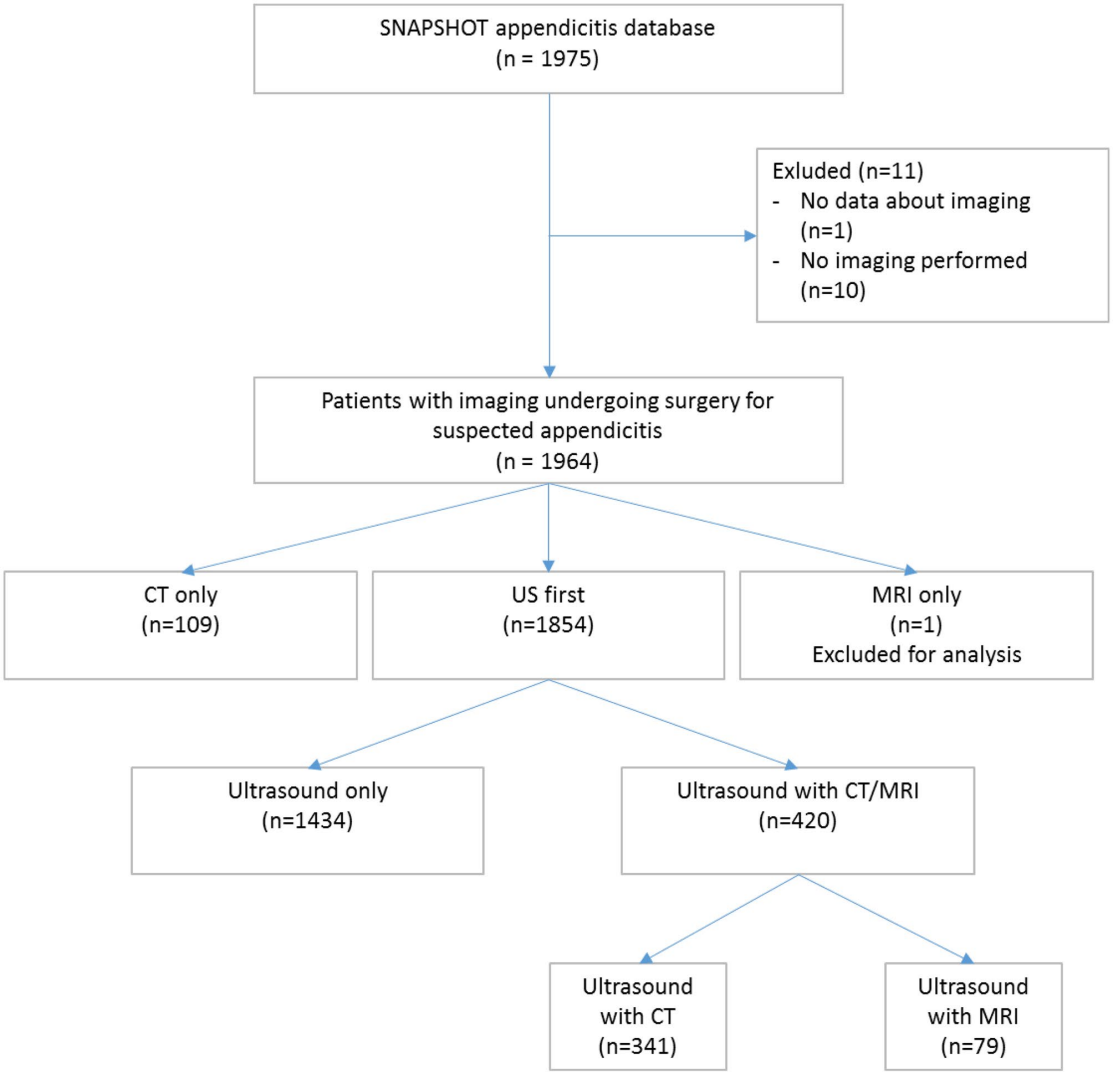
Flowchart

Out of the 1975 patients, a total of 1964 patients were used for this study, as in one patient imaging data were missing, and in ten patients, no imaging was performed. Of 1964 patients, 1807 had appendicitis, of which 617 had complicated appendicitis, 1190 had uncomplicated appendicitis and 157 patients did not have appendicitis according to the surgeon or pathologist. Of these 157 patients without appendicitis, in 99 cases no appendectomy was performed or an uninflamed appendix was found, 36 patients had a neoplasm (either benign or malignant) and 22 patients had another diagnosis (e.g. Crohn’s disease or endometriosis). In 1434 patients (73%), US was used without conditional imaging. In 341 (17%) patients, US was followed by CT, and in 79 (4%) an US was followed by MRI. In 109 (6%) patients, only CT was used, and one (0.1%) patient had an MRI without an US (see flowchart, Fig. 1).

One thousand fifteen (52%) patients were male and 535 (27.2%) were aged < 18 years. No data were missing for age and sex. For BMI, data was missing in 748 (49%) patients older than 16. For patients with a BMI < 25, US as the only modality was performed in 74% compared to 56% of patients with a BMI ≥ 25, (see Table 1). In 91% of children, US was the only imaging modality used versus 66% of adults. According to radiology reports, 314 patients (16%) were labelled as complicated appendicitis and 1526 patients (77%) as uncomplicated appendicitis; imaging was inconclusive in 124 cases (6%).

**Table 1:** Performed imaging modality per subgroup

	US only	CT only	US + CT/MRI*	None
Performed workup	73% (1434/1974)	6% (109/1974)	21% (421/1974)	1% (10/1974)
Age				
< 18 years	91% (490/541)	0% (0/541)	8% (45/541)	1% (6/541)
≥ 18 years	66% (944/1433)	8% (109/1433)	26% (376/1433)	0% (4/1433)
BMI				
BMI < 25	74% (325/438)	4% (19/438)	21% (94/438)	0% (0/438)
BMI ≥ 25	56% (187/333)	11% (37/333)	32% (107/333)	1% (2/333)
Sex				
Male	76% (771/1021)	4% (44/1021)	20% (200/1021)	1% (6/1021)
Female	70% (663/953)	7% (65/953)	23% (221/953)	0% (4/953)
Final diagnosis				
Complicated appendicitis	62% (386/620)	9% (58/620)	28% (173/620)	1% (3/620)
Uncomplicated appendicitis	79% (944/1196)	4% (42/1196)	17% (204/1196)	0% (0/1196)
No appendicitis	66% (104/158)	6% (9/158)	28% (44/158)	0% (0/158)

^*^Conditional CT/MRI (CT only after negative or inconclusive US, as according to national appendicitis guideline)

### Outcomes

#### Overall workup

Overall, 1840 (94%) of all patients with suspected acute appendicitis had a conclusive result based on imaging. The other 124 (6%) patients were operated with inconclusive imaging. Of 1807 patients with the final diagnosis of acute appendicitis, the radiological diagnosis was appendicitis in 1714 (94.7%) cases.

The sensitivity and specificity of the workup as performed for discriminating complicated from uncomplicated appendicitis were 35% (213/617) and 93% (1246/1347), respectively (see Table 2); PPV for complicated appendicitis was only 68% and NPV 76%. Sensitivity and specificity were comparable if the reference standard was defined by the surgeon only or pathologist only (Table S1 and S2). For any given imaging workup, sensitivity, specificity, PPV and NPV for complicated appendicitis were not significantly different in age, sex and BMI (see Table 3).

**Table 2:** Diagnostic accuracy for complicated appendicitis according to performed imaging workup

	Sensitivity	Specificity	PPV	NPV
Overall	35% (213/617)	93% (1246/1347)	68% (213/314)	76% (1246/1650)
US	32% (122/386)	94% (926/1048)	65% (122/187)	79% (926/1247)
CT	45% (26/58)	88% (45/51)	81% (26/32)	58% (45/77)
US + CT/MRI*	38% (65/173)	88% (218/248)	68% (65/95)	67% (218/326)

*PPV* positive predictive value, *NPV* negative predictive value

*Conditional CT/MRI (CT only after negative or inconclusive US, as according to national appendicitis guideline)

**Table 3:** Diagnostic accuracy for complicated appendicitis for subgroups after imaging workup

	Sensitivity	Specificity	PPV	NPV	*P*-value
Age					0.15
< 18 years	39% (59/150)	94% (362/385)	72% (59/82)	80% (362/453)	
≥ 18 years	33% (154/467)	92% (884/962)	66% (154/232)	74% (884/1197)	
BMI					0.45
BMI < 25	30% (40/133)	94% (286/305)	68% (40/59)	75% (286/379)	
BMI ≥ 25	30% (35/117)	92% (197/279)	67% (35/52)	73% (197/279)	
Sex					0.09
Male	32% (103/327)	92% (633/688)	65% (103/158)	74% (633/857)	
Female	38% (110/290)	93% (613/659)	71% (110/156)	77% (613/793)	

The *p*-value was calculated by chi-square test for sensitivity and specificity. Only the lowest value was mentioned

*PPV* positive predictive value, *NPV* negative predictive value

#### Ultrasound

In 1854 patients, ultrasound was the modality of the first choice, 1706 patients had appendicitis and 148 had an alternative diagnosis. In 420 of 1854 (22.7%) cases, US was inconclusive or negative, and an additional CT or MRI was performed. In 1434 patients, US was performed without additional imaging. In 84 of 1854 (4.5%) cases, US was inconclusive, but patients went for surgery without any further imaging. In 386 of 1434, complicated appendicitis was the final diagnosis, in 944 uncomplicated and in 104 cases the final diagnosis was other than appendicitis. The sensitivity of US for complicated appendicitis was 34% (122/386) and specificity 94% (983/1048). Diagnostic accuracy was higher in children than adults; sensitivity was 41.2% vs. 26.4% and specificity 94.6% vs. 93.4%, respectively, *p* = 0.003. For age, sex and BMI, no significant differences in imaging performance were found, see Table S1.

#### CT

In 109 patients, only CT was performed. Of these, 100 patients had a final diagnose of acute appendicitis. Ninety-six percent (96/100) of patients operated for acute appendicitis were correctly diagnosed with CT only.

In 58 patients, the final diagnosis was complicated appendicitis, in 42 uncomplicated appendicitis and in 9 patients no appendicitis. Sensitivity and specificity for complicated appendicitis, in patients who underwent CT only, were 45% (26/58) and 88% (45/51), respectively. No significant differences were found for the subgroups age, BMI or sex, see Table S1.

### US with conditional CT or MRI

In 420 cases, US was inconclusive, and an additional CT or MRI was performed. Of these, 376 patients did have acute appendicitis. Ninety-four percent (353/376) of patients operated for acute appendicitis were correctly diagnosed with US and conditional CT or MRI. In 172 patients, the final diagnosis was complicated appendicitis. Sensitivity and specificity for complicated appendicitis were 37% (64/172) and 88% (218/248), respectively. No significant differences were found in the subgroups age, BMI and sex, see Table S1.

## Discussion

Given current imaging workup, on the whole, following the national guideline, 94.7% of patients selected for appendectomy with the final clinical and imaging diagnosis of acute appendicitis had a correct diagnosis of appendicitis. Discriminating complicated appendicitis from uncomplicated appendicitis by imaging workup showed poor results with a sensitivity of 35%, although specificity was 93%. The highest sensitivity (45%) and positive predictive value (81%) for complicated appendicitis were accomplished by a CT scan only approach. For relevant subgroups such as age, sex and BMI, no clinically relevant differences in discriminatory performance of the imaging modalities were found.

A prospective study exploring the diagnostic accuracy of imaging for perforated appendicitis has found a sensitivity and specificity of 55% and 88%, respectively [17]. Another prospective study (OPTIMAP study) describes diagnostic accuracy results for US with conditional CT if necessary and compares these with MRI alone. The results of that study are largely in line with the present study, finding a sensitivity and specificity for complicated appendicitis for US with conditional CT of 48% and 93% and for MRI alone 57% and 86%, respectively [12]. However, we found lower sensitivities in diagnosing complicated appendicitis. This difference may be explained by research bias in former studies. Radiologists in the present study did not know that their reports would be checked and reports were not standardized. Present findings, therefore, represent real-world data of radiological results of patients with suspected appendicitis.

Routine workup with ultrasound combined with MRI or CT, if necessary, is therefore an excellent discriminator between appendicitis and another abdominal disease. In diagnosing acute appendicitis, recent literature shows a pooled sensitivity and specificity for US of 69% (95% CI 59–78%) and 81% (95% CI 73–88%), respectively [18]. For CT, pooled sensitivity and specificity is 91% (95%CI 84–95%) and 90% (95% CI 85–94%) [4], and for MRI, 96.6% (95% CI 92–99%) and 96% (95% CI 89.4%–98.4%) [19](19). In a conditional CT approach, CT follows US in case of negative or inconclusive US, thereby incorporating the limited sensitivity but high specificity of US for appendicitis in an efficient imaging strategy. For US with conditional CT, a sensitivity for acute appendicitis of 97% and specificity of 91% have been reported previously, and for MRI this is 98% and 88%, respectively [20].

In discriminating complicated from uncomplicated appendicitis, results of imaging are poor in both the present study and published literature. Scoring systems, including both clinical and imaging features, perform better in ruling out complicated appendicitis [14, 15]. Atema et al. has constructed two scoring systems (Severity of Appendicitis Systems, SAS), one including clinical and US features for complicated appendicitis and one including clinical and CT features. SAS achieves a sensitivity of 97% and 90%, for US-SAS and CT-SAS respectively, and a specificity of 46 and 70%; negative predictive values are 97.1% and 94.7%, respectively. Avanesov et al. also have developed a scoring system, including both clinical and CT features to exclude complicated appendicitis and found a sensitivity of 82% and specificity of 93% [14]. However, both these scoring systems are not externally validated yet, and more research should be conducted.

## Limitations

Limitations in the current study include that this dataset does not contain all data on true negative patients, i.e. negative imaging results and no appendicitis. Therefore, diagnostic accuracy for the diagnosis of acute appendicitis was not the focus of this study. Importantly, for discriminating complicated from uncomplicated appendicitis, however, contingency Tables could be constructed, as all consecutive patients undergoing appendectomy for the imaging diagnosis of appendicitis were included. The availability of BMI data was limited; however, the proportion of overweight patients as found was comparable to the average Dutch population. Therefore, it was assumed that these missings were at random [21].

Another limitation of the present study is that we were not able to evaluate imaging results based on a dichotomised decision of the radiologist assigning either a complicated or uncomplicated appendicitis label to each patient. Radiology reports were not standardized, and in many cases, did not explicitly further define the diagnosis of acute appendicitis in complicated or uncomplicated appendicitis. Therefore, our results might be biased to some extent by retrospective interpretation of radiological reports, or because of under-registration of signs of complicated appendicitis by radiologists in their reports. On the other hand, the present study accurately reflects daily practice at the Emergency Department as surgeons interpret written reports of radiologists and thereby classify patients (subconsciously) in complicated or uncomplicated appendicitis. Radiology reports were interpreted by local researchers, which might lead to interobserver variability. The major strength of this study is that it represents real-life data results. In the future, standardised imaging reports might be necessary to investigate the true discriminatory capacity of imaging modalities in differentiating complicated from uncomplicated appendicitis.

## Conclusions

A diagnostic workup with stepwise imaging, using a conditional CT or MRI strategy, poorly discriminates between complicated and uncomplicated appendicitis in daily practice. A CT only approach was not associated with better discriminatory performance.

## Article summary

Why is this topic important?

More research in discriminating complicated from uncomplicated appendicitis is necessary, before conservative treatment for uncomplicated appendicitis is implemented.

What does this study attempt to show?

This study attempts to show the discriminatory capacity of the diagnostic workup with stepwise imaging, using a conditional CT or MRI strategy between complicated and uncomplicated appendicitis in daily practice.

What are the key findings?

A diagnostic workup with stepwise imaging, using a conditional CT or MRI strategy, poorly discriminates between complicated and uncomplicated appendicitis in daily practice.

How is patient care impacted?

Even more research in discriminating complicated from uncomplicated appendicitis is necessary, before conservative treatment for uncomplicated appendicitis is implemented. Imaging alone should not be used to discriminate between complicated and uncomplicated appendicitis.

## Supplementary Information

Below is the link to the electronic supplementary material.

Supplementary file1 (DOCX 14 KB)

## References

[1] Gorter RR, Eker HH, Gorter-Stam MA, Abis GS, Acharya A, Ankersmit M et al (2016) Diagnosis and management of acute appendicitis. EAES consensus development conference 2015. Surg Endosc 30(11):4668–90 10.1007/s00464-016-5245-7 PMC5082605 27660247

[2] Dutch Society for Surgery NvvH (2019) Richtlijn Acute Appendicitis (Dutch Guideline Acute Appendicitis) [Guideline]. Available from: https://richtlijnendatabase.nl/richtlijn/acute_appendicitis/startpagina_-_acute_appendicitis.html. Accessed Date 01-01-2022

[3] Smith MP, Katz DS, Lalani T, Carucci LR, Cash BD, Kim DH (2015). ACR appropriateness criteria(R) right lower quadrant pain–suspected appendicitis. Ultrasound Q.

[4] van Randen A, Bipat S, Zwinderman AH, Ubbink DT, Stoker J, Boermeester MA (2008). Acute appendicitis: meta-analysis of diagnostic performance of CT and graded compression US related to prevalence of disease. Radiology.

[5] Livingston EH (2007) Disconnect between incidence of nonperforated and perforated appendicitis: implications for pathophysiology and management. Ann Surg 245 10.1097/01.sla.0000256391.05233.aa PMC1876946 17522514

[6] Bhangu A (2015) Acute appendicitis: modern understanding of pathogenesis, diagnosis, and management. Lancet (London, England) Sep 26;386(10000):1278–1287 10.1016/S0140-6736(15)00275-5 26460662

[7] Rollins KE, Varadhan KK, Neal KR, Lobo DN (2016). Antibiotics versus appendicectomy for the treatment of uncomplicated acute appendicitis: an updated meta-analysis of randomised controlled trials. World J Surg.

[8] Sallinen V, Akl EA, You JJ, Agarwal A, Shoucair S, Vandvik PO (2016). Meta-analysis of antibiotics versus appendicectomy for non-perforated acute appendicitis. Br J Surg.

[9] Flum DR, Davidson GH, Monsell SE, Shapiro NI, Odom SR, Sanchez SE (2020). A randomized trial comparing antibiotics with appendectomy for appendicitis. N Engl J Med.

[10] Salminen P, Tuominen R, Paajanen H, Rautio T, Nordstrom P, Aarnio M (2018). Five-year follow-up of antibiotic therapy for uncomplicated acute appendicitis in the APPAC randomized clinical trial. JAMA.

[11] NvvH (2018) Richtlijn Beleid rondom Spoedoperaties [Guideline]. Available from: https://richtlijnendatabase.nl/richtlijn/beleid_rondom_spoedoperaties/startpagina_-_beleid_rondom_spoedoperaties.html. Accessed Date 01-01-2022

[12] Leeuwenburgh MM, Wiezer MJ, Wiarda BM, Bouma WH, Phoa SS, Stockmann HB (2014). Accuracy of MRI compared with ultrasound imaging and selective use of CT to discriminate simple from perforated appendicitis. Br J Surg.

[13] Kim HY, Park JH, Lee YJ, Lee SS, Jeon JJ, Lee KH (2017) Systematic review and meta-analysis of CT features for differentiating complicated and uncomplicated appendicitis. Radiology 171260 10.1148/radiol.2017171260 29173071

[14] Avanesov M, Wiese NJ, Karul M, Guerreiro H, Keller S, Busch P (2018). Diagnostic prediction of complicated appendicitis by combined clinical and radiological appendicitis severity index (APSI). Eur Radiol.

[15] Atema JJ, van Rossem CC, Leeuwenburgh MM, Stoker J, Boermeester MA (2015). Scoring system to distinguish uncomplicated from complicated acute appendicitis. Br J Surg.

[16] van Rossem CC, Bolmers MD, Schreinemacher MH, van Geloven AA, Bemelman WA (2016). Prospective nationwide outcome audit of surgery for suspected acute appendicitis. Br J Surg.

[17] Kim K, Kim YH, Kim SY, Kim S, Lee YJ, Kim KP (2012). Low-dose abdominal CT for evaluating suspected appendicitis. N Engl J Med.

[18] Giljaca V, Nadarevic T, Poropat G, Nadarevic VS, Stimac D (2017). Diagnostic accuracy of abdominal ultrasound for diagnosis of acute appendicitis: systematic review and meta-analysis. World J Surg.

[19] Repplinger MD, Levy JF, Peethumnongsin E, Gussick ME, Svenson JE, Golden SK (2016). Systematic review and meta-analysis of the accuracy of MRI to diagnose appendicitis in the general population. J Magn Reson Imaging.

[20] Leeuwenburgh MM, Wiarda BM, Wiezer MJ, Vrouenraets BC, Gratama JW, Spilt A (2013). Comparison of imaging strategies with conditional contrast-enhanced CT and unenhanced MR imaging in patients suspected of having appendicitis: a multicenter diagnostic performance study. Radiology.

[21] Zantinge EM, Wilk EAvd (2020) Overgewicht (RIVM). Available from: https://www.volksgezondheidenzorg.info/onderwerp/overgewicht/cijfers-context/huidige-situatie#definities. Accessed Date 01-01-2022

