# Supplementary material for: Accuracy of imaging in discriminating complicated from uncomplicated appendicitis in daily clinical practice
Source: Int J Colorectal Dis. 2022 May 18;37(6):1385–91. doi: 10.1007/s00384-022-04173-z (PMC9167165; doi:10.1007/s00384-022-04173-z)
Supplement: Supplementary file 1 — Supplementary file1 (DOCX 14 KB) [file 384_2022_4173_MOESM1_ESM.docx]

Table S1, diagnostic accuracy for complicated appendicitis according to performed imaging workup with the peroperative findings by surgeon only as reference standard

|  | Sensitivity | Specificity | PPV | NPV |
| --- | --- | --- | --- | --- |
| Overall | 35% (223/638) | 93% (1235/1326) | 71% (223/314) | 75% (1235/1650ss) |
| US | 32% (124/394) | 94% (977/1040) | 66% (124/187) | 78% (977/1247) |
| CT | 44% (27/61) | 90% (43/48) | 84% (27/32 | 56% (43/77) |
| US+CT/MRI* | 40% (72/183) | 90% (215/238) | 76% (72/95) | 66% (215/326) |

*PPV, Positive Predictive Value; NPV, Negative Predictive Value*
* Conditional CT/MRI (CT only after negative or inconclusive US, as according to national appendicitis guideline)

Table S2, diagnostic accuracy for complicated appendicitis according to performed imaging workup with histopathalogical findings by pathologist only as reference standard

|  | Sensitivity | Specificity | PPV | NPV |
| --- | --- | --- | --- | --- |
| Overall | 34% (126/373) | 88% (1403/1591) | 40% (126/314) | 85% (1403/1650) |
| US | 31% (70/228) | 90% (1089/1206) | 37% (70/187) | 87% (1089/1247) |
| CT | 40% (16/40) | 77% (53/69) | 50% (16/32) | 69% (53/77) |
| US+CT/MRI* | 38% (40/104) | 83% (261/316) | 42% (40/95) | 80% (261/326) |

*PPV, Positive Predictive Value; NPV, Negative Predictive Value*
* Conditional CT/MRI (CT only after negative or inconclusive US, as according to national appendicitis guideline)

Table S1, diagnostic accuracy for complicated appendicitis for subgroups per imaging modality.

|  |  | Sensitivity | Specificity | p-value |
| --- | --- | --- | --- | --- |
| **Ultrasound** | Age  - < 18 years  - ≥ 18 years | 41% (56/136)  26% (66/250) | 95% (335/354)  93% (648/694) | 0.003 |
|  | BMI  - BMI < 25  - BMI ≥ 25 | 29% (22/76)  27% (15/56) | 96% (240/249)  95% (124/131) | 0.43 |
|  | Sex  - Male  - Female | 28% (63/223)  36% (59/163) | 93% (511/548)  94% (472/500) | 0.10 |
| **CT only** | Age  - < 18 years  - ≥ 18 years | -  45% (26/58) | -  88% (45/51) | - |
|  | BMI  - BMI < 25  - BMI ≥ 25 | 46% (5/11)  53% (9/17) | 100% (8/8)  85% (17/20) | 0.54† |
|  | Sex  - Male  - Female | 48% (10/21)  43% (16/37) | 83% (19/23)  93% (26/28) | 0.39† |
| **US+CT/MRI*** | Age  - < 18 years  - ≥ 18 years | 15% (2/13)  39% (62/159) | 87% (27/31)  88% (191/217) | 0.14† |
|  | BMI  - BMI < 25  - BMI ≥ 25 | 28% (13/46)  25% (11/44) | 79% (38/48)  89% (56/63) | 0.16 |
|  | Sex  - Male  - Female | 35% (29/82)  39% (35/90) | 88% (103/117)  88% (115/131) | 0.63 |

*PPV, Positive Predictive Value; NPV, Negative Predictive Value*. The p-value was calculated by chi-square test for sensitivity and specificity. Only the lowest value was mentioned. * Conditional CT/MRI (CT only after negative or inconclusive US, as according to national appendicitis guideline) † Fisher Exact test was used
